# Gastrointestinal toxicities of proteasome inhibitor therapy

**DOI:** 10.1007/s00432-024-05716-3

**Published:** 2024-07-05

**Authors:** Jay Shah, Samanthika Devalaraju, Elliot Baerman, Irene Jeong-Ah Lee, Kei Takigawa, Antonio Pizuorno Machado, Christine Catinis, Malek Shatila, Krishnavathana Varatharajalu, Mehnaz Shafi, Hans C. Lee, Paolo Strati, Anusha Thomas, Yinghong Wang

**Affiliations:** 1 Department of Internal Medicine, Baylor College of Medicine, Houston, TX USA; 2 Department of Internal Medicine, The University of Texas Health Science Center, Houston, TX USA; 3 Department of Gastroenterology, Hepatology, and Nutrition, The University of Texas MD Anderson Cancer Center, 1515 Holcombe Blvd., Unit 1466, Houston, TX 77030 USA; 4 Department of Lymphoma-Myeloma, The University of Texas MD Anderson Cancer Center, Houston, TX USA

**Keywords:** Proteasome inhibitors, Gastrointestinal toxicity, Multiple myeloma, Bortezomib, Carfilzomib, Ixazomib

## Abstract

**Purpose:**

Proteasome inhibitors (PIs), which cause cell death via tumor suppressor and pro-apoptotic proteins, are integral to treatment of many hematologic malignancies but are limited by their gastrointestinal adverse effects. Evidence regarding these PI-related adverse effects is scant. In this study, we evaluated gastrointestinal adverse events caused by PIs and compared gastrointestinal toxicities between bortezomib, carfilzomib, and ixazomib.

**Methods:**

We conducted a retrospective study of cancer patients treated with PIs at a tertiary care cancer center to investigate the clinical characteristics of PI-related gastrointestinal adverse events.

**Results:**

Our sample comprised 973 patients with PI exposure and stool studies ordered between January 2017 and December 2022. Of these, 193 patients (20%) had PI-related gastrointestinal toxicity based on clinical symptoms and stool study results. The most common symptom was diarrhea, present in 169 (88% of those with gastrointestinal toxicity). Twenty-two (11%) required hospitalization, and 71 (37%) developed recurrence of symptoms. Compared to bortezomib or carfilzomib, ixazomib had a longer interval from PI initiation to the onset of gastrointestinal symptoms (313 days vs 58 days vs 89 days, *p* = 0.002) and a significantly lower percentage of diarrhea-predominant presentation of gastrointestinal toxicity (71% vs 96% vs 91%, *p* = 0.048).

**Conclusion:**

While PI-related gastrointestinal toxicities have various presentations and courses based on different regimens, the vast majority of patients presented with milder disease behavior. Despite a considerably high rate of hospitalization and recurrence after treatment necessitating optimization of clinical management, our cohort demonstrates favorable outcomes without long-term consequences.

**Supplementary Information:**

The online version contains supplementary material available at 10.1007/s00432-024-05716-3.

## Introduction

Eukaryotic cells utilize the ubiquitin–proteasome pathway to maintain homeostasis. This intracellular protein degradation pathway mediates complex functions necessary for cellular survival, including apoptosis, DNA repair, and cell cycle progression (Manasanch and Orlowski 2017). Proteasome inhibition can lead to cell death through the accumulation of tumor suppressor and pro-apoptotic proteins, making this pathway an appealing therapeutic target for new oncologic therapies (Orlowski and Baldwin 2002; Lu and Hunter 2010; Love et al. 2013). The development of proteasome inhibitors (PIs), such as bortezomib, carfilzomib, and ixazomib, has provided a breakthrough in the management of hematologic malignancies, and these agents are now a treatment option for multiple myeloma and mantle cell lymphoma (Manasanch and Orlowski 2017; Stansborough and Gibson 2017).

Numerous studies published over the past two decades demonstrate improved patient outcomes and increased progression-free survival in multiple myeloma patients treated with PIs (Stansborough and Gibson 2017; Richardson et al. 2003; Moreau et al. 2011, 2016; Dimopoulos et al. 2016). Despite these improvements, a limiting factor in the utility of these drugs remains their adverse effect profile, which includes peripheral neuropathy, fatigue, cytopenias, heart failure, and gastrointestinal (GI) toxicity (Manasanch and Orlowski 2017; Stansborough and Gibson 2017). A multicenter phase 2 trial examining the use of intravenous bortezomib, the first PI approved for clinical use in patients with relapsed or refractory myeloma, reported significant incidence of peripheral neuropathy and GI toxicity, most notably nausea, vomiting, diarrhea, and constipation (Stansborough and Gibson 2017; Richardson et al. 2003; Muz et al. 2016). To improve the efficacy and tolerability of these agents, scientists have developed new routes of administration as well as newer generations of PIs. Carfilzomib is an irreversible epoxyketone-based second-generation PI developed for bortezomib-refractory multiple myeloma. Ixazomib is a newer, oral formulation of PI therapy with a chemically distinct molecular structure from carfilzomib and bortezomib (Moreau et al. 2016; Kumar et al. 2014). Like bortezomib, ixazomib is a reversible boronic acid PI, but it has more specific binding to the 20S proteasome subunit with the goal of a more tolerable adverse effect profile than bortezomib (Manasanch and Orlowski 2017). Oprozomib, delanzomib, and marizomib are other PIs currently being investigated for potential anticancer uses (Narayanan et al. 2020). Further drug information can be found in Supplemental Table 1. There is still scant evidence regarding the GI adverse effect profiles of different PI agents (Moreau et al. 2016).

Despite their associated toxicities, PI agents remain an integral component of the chemotherapeutic regimens of patients with multiple myeloma because these agents are associated with improvement in patient outcomes. The underlying mechanism of PI-associated GI toxicity remains unknown, making it an important area of study. To better understand the characteristics of GI toxicity in patients treated with these agents, we conducted a retrospective study among cancer patients at a tertiary care center to investigate the features, predispositions, incidences, and severity of GI-related symptoms among patients receiving different PI regimens. We sought to identify clinical and, where available, endoscopic and histologic features correlated with PI-associated GI toxicity to help prevent or reduce the severity of this phenomenon.

## Methods

### Patient selection

**Fig. 1:**
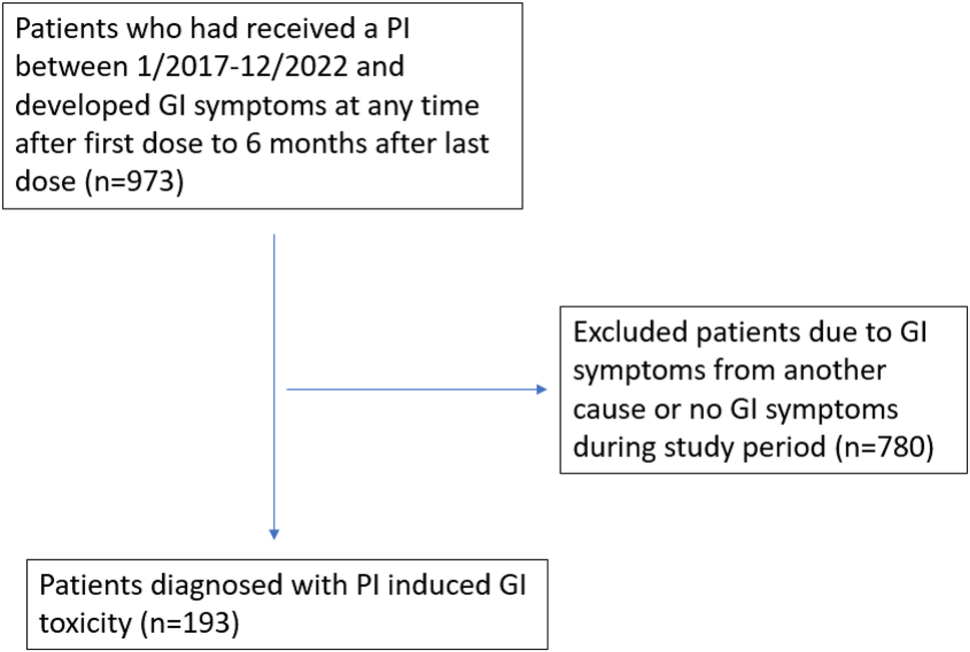
Patient selection flow chart

This single-center retrospective study was approved by the Institutional Review Board of the participating institution. First, we extracted the records of patients at our tertiary care center who had (1) a diagnosis of cancer, (2) received a PI during January 2017 through December 2022, and (3) developed GI symptoms requiring stool study work-up at any point between their initial dose and 6 months after their last dose of PI were extracted. The PI agents included were bortezomib, carfilzomib, ixazomib, oprozomib, delanzomib, and marizomib. Patients were included in the study if expert opinion in the medical record indicated a link between the onset of symptoms and PI use with stool studies performed, if other potential causes such as active GI infection or laxative overuse were ruled out, and if a clear temporal relationship between PI use and GI symptoms was established. Patients who had a diagnosis of inflammatory bowel disease (IBD) or microscopic colitis prior to treatment, who had active symptoms of GI toxicity at time of PI initiation, or who had clear evidence of GI toxicity from another cause, such as irritable bowel syndrome (IBS), laxative overuse, or active GI infection, during time of GI toxicity were excluded. The patients were assessed by an initial reviewer, and if included were assessed by a second reviewer to confirm eligibility. If the second reviewer identified a patient who met any exclusion criteria, the patient was removed from the analysis. Figure 1 details the selection process.

### Data collection

All patient data were collected through institutional electronic medical record databases. We recorded basic demographic data; cancer features; PI therapy used; GI toxicity characteristics, diagnosis, and treatment; and outcome data. The demographic data included age, gender, comorbidities, and diagnosis of a non-GI immune-related adverse event. Cancer data included cancer type and stage at PI initiation. PI data included name, date of first dose, and date of last dose before stopping or holding due to GI toxicity. GI toxicity characteristics included the date of diagnosis, duration of symptoms, and severity of diarrhea and colitis (graded based on the National Cancer Institute’s Common Terminology Criteria for Adverse Events version 5.0). Diagnostic information included fecal lactoferrin, fecal calprotectin, and endoscopy data. GI toxicity treatment type, duration, and doses were recorded. Both short-term outcomes, such as hospitalization and length of stay, as well as long-term outcomes, such as recurrence, complications, and date of death or last follow-up, were recorded.

### Statistical analysis

SPSS version 29 software was used for data analysis and visualization. The distribution of continuous variables was summarized by their median and interquartile range (IQR). The distribution of categorical variables was summarized in terms of their frequencies and percentages. One-way analysis of variance testing was used for continuous variables and Pearson’s chi-squared test was used for categorical variables related to patient characteristics.

## Results

### Patient characteristics

Among the 973 patients meeting the study criteria, 193 were deemed to have PI-associated GI adverse events (19.8%) (Fig. 1). Of these 193 patients, the median age was 65 (IQR 56–70). A total of 103 (53.3%) were male and 145 (75.1%) were white. The median Eastern Cooperative Oncology Group performance status was 1 (IQR 1–2). 187 (96.9%) had a hematologic malignancy. These included 181 (93.8%) with multiple myeloma, 3 (1.6%) with lymphoma, 2 (1.0%) with systemic amyloidosis, and 1 (0.5%) with leukemia. Bortezomib was the most common PI used, received by 114 (59.1%) patients, followed by 55 (28.5%) receiving carfilzomib and 24 (12.4%) receiving ixazomib. No patients received oprozomib, delanzomib, or marizomib during the study period. The median follow-up duration was 1.3 years (IQR 0.4–3.3 years) with all-cause mortality in 90 (46.6%) patients. Further details are available in Table 1. Supplemental Table 2 details patient characteristics in subgroup of patients diagnosed with multiple myeloma specifically.

**Table 1:** Patient demographics, *n* = 193

Characteristic	No. (%)
Age, median (IQR)	64 (56–70)
Sex: Male	103 (53.3%)
Race: White	145 (75.1%)
ECOG, median (IQR)	1 (1–2)
Cancer type	
Melanoma	1 (0.5%)
Genitourinary cancer	3 (1.6%)
Gastrointestinal cancer	2 (1.0%)
Hematologic cancer	187 (96.9%)
PI used for cancer treatment	
Bortezomib	114 (59.1%)
Carfilzomib	55 (28.5%)
Ixazomib	24 (12.4%)
All-cause mortality	90 (46.6%)
Follow-up duration, years	1.3 (0.4–3.3)

ECOG, Eastern Cooperative Oncology Group performance status; IQR, interquartile range

### GI adverse event rates and severity

Ixazomib had a longer median time to onset of GI symptoms compared to bortezomib (58.3 vs 312.5 days, *p* ≤ 0.05). There was no significant difference in time to symptom onset between ixazomib and carfilzomib or between bortezomib and carfilzomib. The PIs had comparable rates of non-GI immune-related adverse events (range 32.5–41.7%). The PIs had comparable rates of use of concurrent cancer treatment medications (81.8–49.3%). 144 patients (74.6%) received autologous stem cell transplant (ASCT), 83 (43%) before and 61 (32%) after the diagnosis of PI-induced GI toxicity. Diarrhea was the predominant symptom in all three drugs (range 70.8–90.9%), and most cases were grade 1–2 (range 84.9–94.6%). Ixazomib had a higher rate of nausea or vomiting compared to bortezomib (37.5% vs 6.1%, *p* ≤ 0.05). Rates of constipation (range 0–7%), blood in stool (range 0–3.6%), and abdominal pain (range 2.6–8.3%) were similar between drugs. Further details are available in Table 2. Supplemental Table 3 details GI toxicity in subgroup of patients diagnosed with multiple myeloma specifically. The rates of lower GI toxicity in the literature for PI-based regimens range from 30 to 36% (Supplemental Table 4).

**Table 2:** Patient clinical characteristics, *n* = 193

Characteristic	Bortezomib *n* = 114	Carfilzomib *n* = 55	Ixazomib *n* = 24
	No. (%)	No. (%)	No. (%)
Time from PI initiation to symptom onset, days, median (IQR)	58.3 (17.1–148.4)^a^	89 (31–193)	312.5 (42.5–835.5)^a^
Other irAEs	37 (32.5%)	18 (32.7%)	10 (41.7%)
Concurrent cancer medications^b^	71 (49.3%)	45 (81.8%)	16 (66.7%)
Location of GI toxicity			
Upper GI	9 (7.9%)	7 (12.7%)	9 (37.5%)*
Lower GI	109 (95.6%)	50 (90.9%)	16 (66.7%)*
Hepatobiliary	1 (0.9%)	1 (1.8%)	1 (4.2%)
Pancreatic	–	–	–
Presenting symptoms			
Nausea/vomiting	7 (6.1%)^a^	9 (16.4%)	9 (37.5%)^a^
Diarrhea	102 (89.5%)^a^	50 (90.9%)	17 (70.8%)^a^
Constipation	8 (7.0%)	1 (1.8%)	0 (0%)
Blood in stool	3 (2.6%)	2 (3.6%)	0 (0%)
Abdominal pain	3 (2.6%)	4 (7.3%)	2 (8.3%)
Peak diarrhea CTCAE grade			
1	24 (45.3%)	19 (51.4%)	10 (58.8%)
2	21 (39.6%)	16 (43.2%)	6 (35.3%)
3	7 (13.2%)	2 (5.4%)	1 (5.9%)
4	1 (1.9%)	0 (0%)	0 (0%)
Fecal lactoferrin positive, *n* = 7 tested	2 (40.0%)	1 (100%)	1 (100%)
Peak fecal calprotectin values, mean ± SEM (*n* = 8)	30.5 ± 8.9 (*n* = 6)	57.3 (*n* = 1)	31 (*n* = 1)

CTCAE, Common Terminology Criteria for Adverse Events; GI, gastrointestinal; irAE, immune-related adverse event; IQR, interquartile range; PI, proteasome inhibitor; SEM, standard error of the mean

*This group differed significantly from the other two groups at the *p* < 0.05 level

^a^These two groups differed significantly at the *p* < 0.05 level (*p* = 0.01)

^b^Included patients taking cyclophosphamide, daratumumab, isatuximab, lenalidomide, or pomalidomide. Only 1 (0.5%) patient developed GI Graft-versus-host disease prior to the diagnosis of PI-induced GI toxicity

### Fecal sample and endoscopy findings

Fecal lactoferrin testing was performed in 7 patients, with 4 having positive results. Of these patients with positive lactoferrin results, 2 received bortezomib, 1 received carfilzomib, and 1 received ixazomib. The 3 patients with negative results received bortezomib. Fecal calprotectin testing was performed in 8 patients, wherein the majority (*n* = 7) had normal levels reported (6 patients given bortezomib and 1 given ixazomib). One patient taking carfilzomib had a mildly elevated fecal calprotectin level (57.3 μg/g). Endoscopy was performed in 17 patients (8.8%); 11 (65%) had normal findings, 3 (18%) had non-ulcerative inflammation, and 3 (18%) had ulcerous inflammation, of whom 2 had high-risk endoscopic features. Histologic findings were assessed in 13 (6.7%) patients; 10 (77%) patients had normal findings, 2 (15%) had acute inflammation, and 1 (7.7%) had chronic inflammation. Two patients were tested for both endoscopy and fecal inflammatory biomarkers; 1 patient had normal colonoscopy and fecal calprotectin and lactoferrin results, and the other patient had normal colonoscopy results with elevated lactoferrin but normal calprotectin. Patients given carfilzomib had higher rates of high-risk inflammatory features compared to bortezomib (0% vs 100%, *p* ≤ 0.05) and lower rates of normal histologic findings (33.3% vs 90.0%, *p* ≤ 0.05).

### Adverse event treatment, clinical course, and outcomes

In all 3 PI groups, the majority of patients were treated with supportive measures, such as intravenous fluids, anti-emetics, and antidiarrheals (79.2–83.3%). No patients in the study received steroids or other immunosuppression for treatment of their GI toxicity. The median length of treatment was 11–12 days for bortezomib and carfilzomib, but longer for ixazomib, at 57 days. Rates of hospitalization for GI symptoms were 8.33–16.4%, and the median length of hospitalization was 3–6.5 days. Treatment response to supportive care for GI symptoms ranged from 84.2 to 93.7%. Zero (0%) patients had a complication, such as perforation or chronic colitis, from their toxicity. Symptom recurrence ranged from 25.0 to 45.5%. 30% of patients stopped PI therapy, 6% had their PI dose reduced, 12% were switched to another PI, 49% continued their therapy without change, and 3% the treatment strategy was unclear. All-cause mortality ranged from 33.3 to 56.4%. Further details are available in Table 3 and Supplemental Table 5.

**Table 3:** Clinical course and outcomes of GI toxicity, *n* = 193

	Bortezomib *N* = 114	Carfilzomib *N* = 55	Ixazomib *N* = 24
Characteristic	No. (%)	No. (%)	No. (%)
Symptom duration, days, median (IQR)	12 (6–30)	10 (5–32)	21.5 (4.5–339.5)
Endoscopy performed	11 (9.6%)	4 (7.3%)	2 (8.3%)
Location of GI toxicity			
Upper GI	0 (0%)	1 (50.0%)	–
Small intestine	3 (100%)	0 (0%)	–
Colon	0 (0%)	1 (50.0%)	–
Gross findings			–
Normal	8 (72.7%)	1 (25.0%)	2 (100%)
Non-ulcerous inflammation	1 (9.1%)	2 (50.0%)	0 (0%)
Ulcerous inflammation	2 (18.2%)	1 (25.0%)	0 (0%)
High-risk features present	0 (0%)^a^	2 (100%)^a^	–
Histologic findings			
Normal	9 (90.0%)^a^	1 (33.3%)^a^	–
Acute inflammation	1 (10.0%)	1 (33.3%)	–
Chronic inflammation	0 (0.0%)	1 (33.3%)	–
Treatment for GI toxicity			
No treatment	19 (16.7%)	12 (21.8%)	5 (20.8%)
Supportive treatment	95 (83.3%)	43 (78.2%)	19 (79.2%)
Duration of treatment, days, median (IQR)	12 (7–32)	11 (6–41)	57 (4–602)
Hospitalization	12 (10.5%)	9 (16.4%)	2 (8.3%)
Duration of hospitalization, days median (IQR)	6.5 (3.5–9)	5 (3–11)	3
Complications from the toxicity	0 (0.0%)	0 (0.0%)	0 (0.0%)
Response, *n* = 157	89 (93.7%)	39 (90.7%)	16 (84.2%)
Recurrence	40 (35.1%)	25 (45.5%)	6 (25.0%)
Mortality	51 (44.7%)	31 (56.4%)	8 (33.3%)

^a^These two groups differed significantly at the *p* < 0.05 level

GI, gastrointestinal; IQR, interquartile range

### OS curves

OS was similar between patients who developed PI-induced GI toxicity and those that did not (p = 0.858) (Supplemental Fig. 1). OS was significantly lower in patients on ixazomib compared to bortezomib and carfilzomib (Supplemental Fig. 2).

## Discussion

PI therapy has provided a breakthrough in the management of hematologic malignancies, but the adverse effect profile remains a concern. There is scant evidence regarding the GI side effect profiles from different PI agents (Moreau et al. 2016). In this study, we demonstrated that among patients who received PI therapy and developed GI symptoms from any cause, PI-induced GI toxicity occurred in less than 20% of patients. The disease course was generally mild and usually resolved with supportive care but was accompanied by a considerable rate of hospitalization and recurrence. Among different PI agents, ixazomib had a significantly delayed onset of GI toxicity and more frequent presentation of upper GI symptoms compared to other PI agents. The majority of patients with PI-induced GI toxicity were able to resume PI treatment of some kind. The presence of PI-induced GI toxicity did not appear to affect survival.

The mechanisms of PI-induced GI toxicity remain a continued area of research. Data from mouse studies have demonstrated an increase in pro-inflammatory cytokine-like TNF-α, interleukin (IL)-1β, and IL-6 in intestinal epithelial cells associated with PI use (Sun et al. 2005; Jannuzzi et al. 2023). Another proposed mechanism is dysregulation of the gut microbiome, though this has not been studied (Alkharabsheh et al. 2020). Further research on the relative risks of PI-induced GI toxicity related to antibiotics and history of abdominal surgery that also indirectly affect the gut microbiome may help elucidate this possible mechanism. Both carfilzomib and ixazomib have been shown in cardiomyocyte models to increase cellular and endoplasmic reticulum stress protein levels (Jannuzzi et al. 2023). A similar effect in the gut would be another potential etiology of GI adverse effects. These proposed mechanisms suggest that anti-inflammatory medications, such as corticosteroids, or fecal microbiota transplants could be potential treatment options for severe or refractory PI-induced GI toxicity. However, such treatment is yet to be studied in the published literature. In our patient population, these treatments were not utilized outside of standard steroid use in treatment regimens, likely because of the high rate of response to supportive care alone. This is in contrast with immunotherapy-induced colitis for which, corticosteroids and biologics have demonstrated high efficacy and become mainstays of treatment (Zou et al. 2021a; Thompson et al. 2022; Schneider et al. 2021; Wang et al. 2018a, b; Halsey et al. 2023). Fecal microbiota transplant has become an emerging treatment for refractory immunotherapy-induced colitis (Wang et al. 2018a, b; Halsey et al. 2023). We speculate that these treatments may similarly be considered in patients with recurrent PI-induced GI toxicity refractory to conservative management. Other than medical treatment, common practice for the higher grade of GI toxicities is to withhold PI treatment until the symptoms are adequately managed and then resume with dose reduction.

PI-induced GI toxicity is a diagnosis of exclusion. A thorough history should be obtained to ensure a temporal relationship between PI use and symptoms and to rule out other etiologies, such as infection, IBD, IBS, and other medication side effects. The evaluation of endoscopy, and fecal inflammatory markers is infrequently used in our population. Fecal calprotectin and lactoferrin are sensitive markers of intestinal inflammation that can aid in the diagnosis of immunotherapy-induced colitis and inflammatory bowel disease (Wang et al. 2018a, b; Som et al. 2019; Zou et al. 2021a, b). In our cohort, of the 15 fecal tests ordered, only 5 (33%) had positive results, suggesting that these markers are not necessarily sensitive tools to capture the mild nature of PI-induced GI toxicity with minimal histologic injury in many cases. It also reflects the practice pattern of underutilization of this non-invasive stool test tool. The low rate of endoscopy evaluation among our cohort suggests the same observation. This pattern contrasts with the management of immunotherapy-induced colitis, where fecal inflammatory markers and endoscopic evaluation have become routine as the most critical tools in prognostic and therapeutic decision-making (Wang et al. 2018a, b; Abu-Sbeih et al. 2018).

Prior comparisons have noted similar GI toxicity profiles between bortezomib and carfilzomib, which are both delivered via an intravenous or subcutaneous route (Stansborough and Gibson 2017). Bortezomib was the first PI approved for use in multiple myeloma (Muz et al. 2016). Carfilzomib is a second-generation PI developed for refractory and relapsed multiple myeloma with a lower incidence of peripheral neuropathy than bortezomib. Ixazomib is a newer, oral formulation of PI therapy that has a chemically distinct molecular structure from carfilzomib and bortezomib (Moreau et al. 2016). While the drugs have not been compared head-to-head, each has been attributed to GI adverse effects, most commonly nausea, vomiting, and diarrhea (Moreau et al. 2016). In the present study, patients given ixazomib took longer to develop GI symptoms, with nausea and vomiting as predominant presentation rather than diarrhea, compared to patients on bortezomib, which may be due to ixazomib’s higher binding specificity to the 20S proteasome subunit. The higher proportion of nausea and vomiting in ixazomib may also be due to its oral pill formulation that requires patients to take it on an empty stomach, which may exacerbate the GI symptoms (Gupta et al. 2016). While more data are still required, patients with a history of diarrhea may be candidates to switch to ixazomib, as there is a case report of a patient who had resolution of diarrhea and associated GI electrolyte losses after switching to ixazomib from carfilzomib (Nakako et al. 2021). The etiology of worse OS in patients on ixazomib is unclear but may have been affected by the small sample size and requires further investigation. While we noted a significantly higher rate of severe colitis on endoscopy in carfilzomib compared to bortezomib, this finding was limited by the small sample size and would require further investigation to determine clinical relevance.

We acknowledge the limitations of this study. The study was designed as a single-center retrospective study. Our diagnostic approach to PI-induced GI toxicity was based on clinical symptoms and required stool tests to be obtained, which may have missed patients who only developed nausea or vomiting or never had stool tests performed without our institution, which may explain our lower rate of GI toxicity compared to the existing clinical trials. No specific clinical test can distinguish PI-induced GI toxicity from other etiologies with complete certainty. Rather, the diagnostic approach has involved a combination of ruling out other etiologies and obtaining a clear history to establish a temporal relationship. Patients who received non-PI cancer treatment in the study window sequentially or concurrently that are composed of certain portion of our study cohort could also have confounded the presentations. Not all the risk factors such as antibiotic use that may play a role in the microbiome alteration and contribute to the onset of GI toxicities were included. The underutilization of endoscopy and fecal inflammatory markers in our cohort limited our opportunity to further clarify the full spectrum of endoscopic and histologic features of PI-related GI toxicity. Finally, certain parameters of GI toxicity were underpowered for comparison between different PI groups for definitive conclusions.

## Conclusion

In patients treated with PIs who developed GI symptoms from any cause, the estimated incidence of PI-induced GI toxicity is less than 20%. Generally, this toxicity has a mild clinical course with favorable response to conservative management, and most patients are able to resume or continue PI therapy. Invasive evaluation such as endoscopy is rarely required. There is considerable variation in clinical presentations of GI toxicity among different PI agents. Rate of hospitalization and symptom recurrence remains concerning, warranting further investigations for better outcomes.

## Supplementary Information

Below is the link to the electronic supplementary material.

Supplementary file1 (DOCX 223 kb)

## Data Availability

Data is provided within the manuscript or supplementary information files.
