# Supplementary material for: Gastrointestinal toxicities of proteasome inhibitor therapy
Source: J Cancer Res Clin Oncol. 2024 Jul 5;150(7):334. doi: 10.1007/s00432-024-05716-3 (PMC11226465; doi:10.1007/s00432-024-05716-3)

| Supplemental Table 1. List of Proteosome Inhibitor Agents | |
| --- | --- |
| Chemical name | Brand name |
| Bortezomib | Velcade, PS-341 |
| Carfilzomib | Kyprolis, CFZ, PR-171 |
| Ixazomib | Ninlaro, IXZ, MLN9708 |
| **Oprozomib** | **ONX 0912, OPZ, PR-047** |
| **Marizomib** | **NPI 0052, salinosporamide A** |
| **Delanzomib** | **CEP-18770** |

| Supplemental Table 2. Patient Demographics with the Diagnosis of Multiple Myeloma, n=181 | |
| --- | --- |
| Characteristic | No. (%) |
| Age, median (IQR) | 65 (57-70) |
| Sex: Male | 94 (52%) |
| Race: White | 122 (67.0%) |
| ECOG, median (IQR) | 1 (1-2) |
| PI used for cancer treatment |  |
| Bortezomib | 108 (60.0%) |
| Carfilzomib | 55 (28.5%) |
| Ixazomib | 18 (9.9%) |
| All-cause mortality | 82 (45%) |
| Follow-up duration, years | 1.4 (0.45-3.4) |

ECOG, Eastern Cooperative Oncology Group performance status; IQR, interquartile range

| Supplemental Table 3. Clinical Characteristics in Patients with the Diagnosis of Multiple Myeloma, n=181 | | | |
| --- | --- | --- | --- |
|  | Bortezomib  n=108 | Carfilzomib  n=55 | Ixazomib  n=18 |
| Characteristic | No. (%) | No. (%) | No. (%) |
| Time from PI initiation to symptom onset, days, median (IQR) | 61 (18.5-155)^a^ | 89 (31-193) | 411 (46-848)^a^ |
| Other irAEs | 34 (31%) | 18 (32.7%) | 6 (33%) |
| Location of GI toxicity |  |  |  |
| Upper GI | 9 (7.9%) | 7 (12.7%) | 8 (44%)* |
| Lower GI | 103 (95%) | 50 (90.9%) | 10 (56%)* |
| Hepatobiliary | 1 (0.9%) | 1 (1.8%) | 1 (5.6%) |
| Pancreatic | 0 (0%) | 0 (0%) | 0 (0%) |
| Presenting symptoms |  |  |  |
| Nausea/vomiting | 7 (6.5%)^a^ | 9 (16.4%) | 8 (44%)^a^ |
| Diarrhea | 98 (91%)^a^ | 50 (90.9%) | 10 (56%)^a^ |
| Constipation | 7 (6.5%) | 1 (1.8%) | 0 (0%) |
| Blood in stool | 3 (2.8%) | 2 (3.6%) | 0 (0%) |
| Abdominal pain | 3 (2.8%) | 4 (7.3%) | 1 (5.6%) |
| Peak diarrhea CTCAE grade |  |  |  |
| 1 | 23 (23.5%) | 19 (51.4%) | 7 (38.9%) |
| 2 | 19 (19.4%) | 16 (43.2%) | 4 (22.2%) |
| 3 | 7 (7.1%) | 2 (5.4%) | 0 (0%) |
| 4 | 1 (1.0%) | 0 (0%) | 0 (0%) |
| Fecal lactoferrin positive, n=6 tested | 2 (40%) | 1 (100%) | 0 (0%) |
| Peak fecal calprotectin values, mean ± SEM (n=7) | 30.5 ± 8.9  (n=6) | 57.3  (n=1) | - |
| ^a^These two groups differed significantly at the p<0.05 level (p=0.01)  *This group differed significantly from the other two groups at the p<0.05 level.  CTCAE, Common Terminology Criteria for Adverse Events; GI, gastrointestinal; irAE, immune-related adverse event; IQR, interquartile range; PI, proteasome inhibitor; SEM, standard error of the mean | | | |

Supplemental Table 4: Incidence of Lower GI Toxicity in PI Regimens from Clinical Trials

| Agents | Incidence of Lower GI toxicity |
| --- | --- |
| DVD, n=498 | 159 (32%)^a^ |
| IRD, n=127 | 44 (35%)^b^ |
| KRD, n=396 | 119 (30%)^c^ |
| IsaKD, n=179 | 64 (36%)^d^ |
| DKD, n=312 | 100 (32%)^e^ |
| ^a^Efficacy and Safety of Daratumumab, Pomalidomide, and Dexamethasone (DPd) Compared to Daratumumab, Bortezomib, and Dexamethasone (DVd) in Daratumumab-Naïve Relapsed Multiple Myeloma. Cancers (Basel). 2023 Oct 9;15(19):4894.  ^b^Survival benefit of ixazomib, lenalidomide and dexamethasone (IRD) over lenalidomide and dexamethasone (Rd) in relapsed and refractory multiple myeloma patients in routine clinical practice. BMC Cancer. 2021 Jan 15;21(1):73.  ^c^Carfilzomib, lenalidomide, and dexamethasone for relapsed multiple myeloma  ^d^Isatuximab, carfilzomib, and dexamethasone in relapsed multiple myeloma (IKEMA): a multicentre, open-label, randomised phase 3 trial. Lancet. 2021 Jun 19;397(10292):2361-2371.  ^e^Carfilzomib, dexamethasone, and daratumumab versus carfilzomib and dexamethasone for patients with relapsed or refractory multiple myeloma. Lancet Oncol. 2022 Jan;23(1):65-76. | |

| Supplemental Table 5. Clinical Course and Outcomes of GI toxicity among Patients with Multiple Myeloma. n=181 | | | |
| --- | --- | --- | --- |
|  | Bortezomib  N=108 | Carfilzomib  N=55 | Ixazomib  N=18 |
| Characteristic | No. (%) | No. (%) | No. (%) |
| Symptom duration, days, median (IQR) | 12 (6-31) | 10 (5-32) | 69(4-430) |
| Endoscopy performed | 11 (9.6%) | 4 (7.3%) | 2 (8.3%) |
| Location of GI toxicity |  |  |  |
| Upper GI | 0 (0%) | 1 (50.0%) | - |
| Small intestine | 3 (100%) | 0 (0%) | - |
| Colon | 0 (0%) | 1 (50.0%) | - |
| Gross findings |  |  | - |
| Normal | 8 (72.7%) | 1 (25.0%) | 2 (100%) |
| Non-ulcerous inflammation | 1 (9.1%) | 2 (50.0%) | 0 (0%) |
| Ulcerous inflammation | 2 (18.2%) | 1 (25.0%) | 0 (0%) |
| High-risk features present | 0 (0%)^a^ | 2 (100%)^a^ | - |
| Histologic findings |  |  |  |
| Normal | 9 (90.0%)^a^ | 1 (33.3%)^a^ | - |
| Acute inflammation | 1 (10.0%) | 1 (33.3%) | - |
| Chronic inflammation | 0 (0.0%) | 1 (33.3%) | - |
| Treatment for GI toxicity |  |  |  |
| No treatment | 15 (13.9%) | 12 (21.8%) | 4 (22.2%) |
| Supportive treatment | 93 (86.1%) | 43 (78.2%) | 14 (77.8%) |
| Duration of treatment, days, median (IQR) | 12 (7-32) | 11 (6-41) | 98.5 (4-733) |
| Hospitalization | 12 (10.5%) | 9 (16.4%) | 2 (8.3%) |
| Duration of hospitalization, days median (IQR) | 6.5 (3.5-9) | 5 (3-11) | 3 |
| Complications from the toxicity | 0 (0.0%) | 0 (0.0%) | 0 (0.0%) |
| Response, | 87 (93.5%) | 39 (90.7%) | 11 (78.6%) |
| Recurrence | 38 (36.9%) | 25 (45.5%) | 6 (33.3%) |
| Mortality | 46 (42.6%) | 31 (56.4%) | 5 (27.8%) |
| ^a^These two groups differed significantly at the p<0.05 level  GI, gastrointestinal; IQR, interquartile range | | | |

**Supplemental Figure 1: Kaplan-Meier Survival Curve for Overall Survival among Patients with and without GI Toxicity**
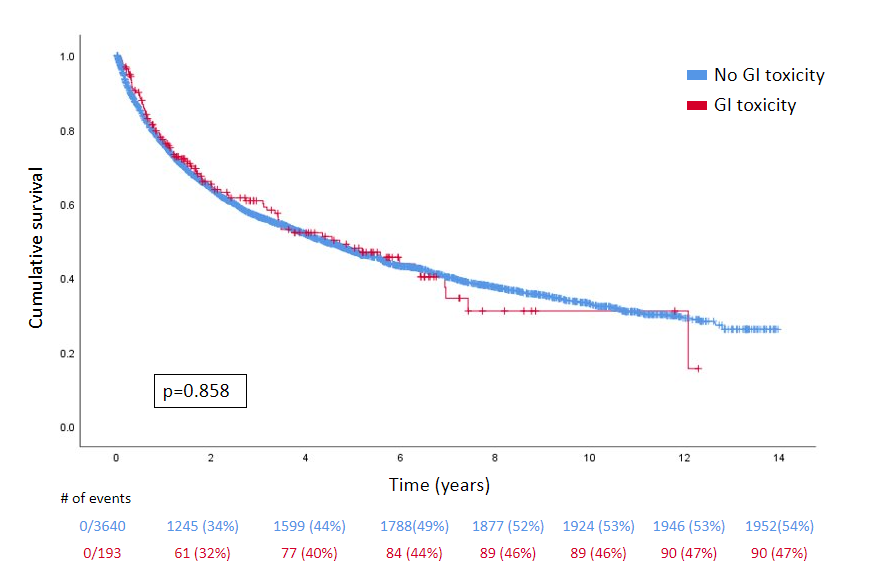


**Supplemental Figure 2: Kaplan-Meier Survival Curve for Overall Survival among Patients with and without GI Toxicity**


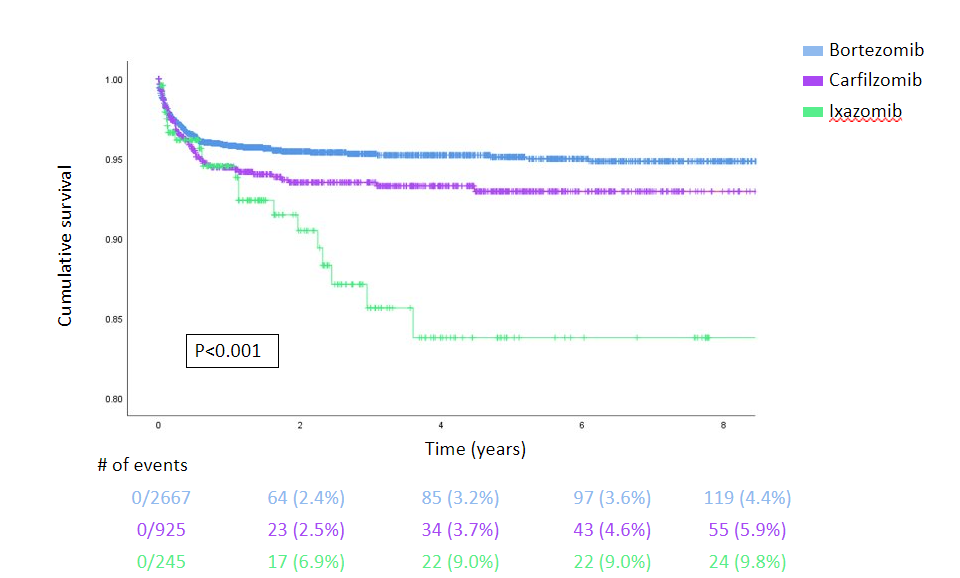

Supplement: Supplementary file 1 — Supplementary file1 (DOCX 223 kb) [file 432_2024_5716_MOESM1_ESM.docx]
